# Value-Added Carp Roe Salad Supplemented with Orange Carrot Pomace Powder

**DOI:** 10.3390/foods14213606

**Published:** 2025-10-23

**Authors:** Roxana Nicoleta Rațu, Genica-Florina Oncică, Florina Stoica, Oana Emilia Constantin, Nicoleta Stănciuc, Iuliana Aprodu, Doina Georgeta Andronoiu, Marija Banožić, Nada Ćujić Nikolić, Gabriela Râpeanu

**Affiliations:** 1 Department of Food Science, Engineering and Applied Biotechnology, Faculty of Food Science and Engineering, Dunarea de Jos University of Galati, 111 Domneasca Street, 800201 Galati, Romania; roxana.ratu@iuls.ro (R.N.R.); genicaoncica@gmail.com (G.-F.O.); oana.constantin@ugal.ro (O.E.C.); nicoleta.stanciuc@ugal.ro (N.S.); iuliana.aprodu@ugal.ro (I.A.); georgeta.andronoiu@ugal.ro (D.G.A.); 2 Faculty of Agriculture, Department of Food Technologies, “Ion Ionescu de la Brad” Iasi University of Life Sciences, 3 Mihail Sadoveanu Alley, 700489 Iasi, Romania; 3 Faculty of Agriculture, Department of Pedotechnics, “Ion Ionescu de la Brad” Iasi University of Life Sciences, 3 Mihail Sadoveanu Alley, 700489 Iasi, Romania; florina.stoica@iuls.ro; 4 Faculty of Agriculture and Food Technology, University of Mostar, Biskupa Čule bb, 88000 Mostar, Bosnia and Herzegovina; marija.banozic@aptf.sum.ba; 5 Institute for Medicinal Plants Research “Dr. Josif Pančić”, Tadeuša Košćuška 1, 11000 Belgrade, Serbia; ncujic@mocbilja.rs

**Keywords:** *Daucus carota*, food by-products, antioxidant activity, carotenoids, nutritional enhancement

## Abstract

Carrot pomace is the solid residue left after juice extraction from carrots. Carrot pomace, typically seen as waste, is gaining recognition for its sustainability and potential to mitigate food waste while offering essential nutrients (phenolics, carotenoids, and β-carotene), which are recognized for their nutraceutical effects and health benefits. A study was conducted to develop a process for creating an innovative product, specifically a carp roe salad with added value, by incorporating carrot pomace. The innovative aspect is represented by using different proportions of carrot powder, 6% and 12%, when creating new varieties of roe salad. The study assesses the impact of carrot pomace powder on the salad’s antioxidant content, physicochemical properties, color, texture, rheological characteristics, and sensory qualities. The value-added products thus obtained are differentiated by superior phytochemical and nutritional characteristics, especially levels of carotenoids (84.01 ± 3.39–111.01 ± 1.68 mg/100 g DW), and the antioxidant activity (550.66 ± 9.25–588.32 ± 9.41 μM TE/g DW) of the developed salad. The obtained products displayed an improved color and texture profile. The sensory evaluation reveals that the carp roe salad with 12% carrot powder was favorably received by consumers, who valued the nuanced changes in flavor and the improved coloration of the product. Rich in antioxidants, fibers, and natural colorants, carrot pomace enhances the product’s value by increasing antioxidant activity and positively influencing sensory properties such as color and aroma. This research highlights the potential of using food by-products to create innovative, value-added products with improved health benefits.

## 1. Introduction

The food sector is always changing, as shown by the fact that new products with health benefits for customers are often produced. Incorporating diverse by-products into food can offer a practical economic advantage and significant health benefits due to their nutritional and functional properties [1]. In carrot juice production, up to 50% of the roots provide pomace, typically utilized as animal feed or fertilizer [2]. The carrot (*Daucus carota*) is a root vegetable that is consumed worldwide. Its color ranges from orange to red, purple, white, and yellow. It is a significant source of dietary fibers, carotenoids, and other functional groups, which provide significant health benefits. β-carotene, vitamins (thiamine, riboflavin, folic acid, and vitamin B-complex), and minerals (calcium, magnesium, potassium, phosphorus, and iron) are among the most significant bioactive compounds [3]. Carotenoids, natural pigments such as β-carotene, lycopene, lutein, and zeaxanthin, are major precursors of vitamin A and exhibit strong antioxidant activity, protecting cells from oxidative stress. Their consumption is associated with reduced risks of cancer, cardiovascular disease, and age-related macular degeneration. In addition, carotenoids support immune function, modulate gene expression, and exert anti-inflammatory effects. Therefore, valorizing carotenoids from juiced carrot pulp is of great nutritional and functional importance [4,5].

The food industry produces large amounts of by-products, whose improper disposal can lead to environmental pollution. However, these residues are rich in valuable compounds such as polysaccharides, polyphenols, and carotenoids, which can be recovered for use in functional foods [6]. In Europe, food processing generates approximately 27.94 million kilograms of waste annually [7]. During carrot juice production, 60–70% of the roots are converted into juice, leaving up to 50% as juiced carrot pulp—a by-product often used as fertilizer or animal feed despite its high nutritional value. This pulp contains essential minerals, including potassium (18.6 mg/kg), calcium (3.0 mg/kg), and iron (30.5 mg/kg), as well as structural fibers such as cellulose (51.6%), hemicellulose (12.3%), pectin (3.88%), and lignin (32.1%) [8,9].

Therefore, the by-products from carrot juice extraction represent a promising source of phytochemicals with significant health-promoting potential, suitable for developing natural ingredients for functional foods and nutraceuticals. Valorizing these by-products not only enhances resource efficiency but also reduces production costs and provides additional economic benefits to producers [7]. Carrot pomace has significant colouring qualities as an additive and is extensively utilised in the manufacturing of diverse food products, including dairy products, confections, mayonnaise, extruded snacks, and bakery products [10,11,12,13,14]. The works in the specialized literature have revealed the possibility of valorizing the carrot powder differently. Tanska et al. [8] showed that carrot powder can replace up to 5% wheat flour in wheat bread to improve quality. Surbhi et al. [2] developed a condensed milk product known as “gazrella” that was created with carrot powder and had favorable overall acceptability responses after six months of storage at room temperature. By adding carrot powder, Kırbaş et al. [15] created a gluten-free cake with improved fiber content and acceptable sensory qualities. To make ready-to-eat enlarged snacks rich in fiber from barley flour, Lotfi Shiraz et al. [16] proposed adding 10% carrot powder.

Carrot dietary fiber also plays a significant role in promoting gastrointestinal health. Additionally, the soluble fiber in carrots acts as a prebiotic, feeding beneficial gut bacteria and promoting a healthy gut microbiome. This study seeks to address the gap in prior research concerning the insufficient investigation of incorporating carrot pomace powder as a bioactive component in roe salad, particularly emphasizing the optimization of carrot powder concentration to improve nutritional value and sensory attributes while maintaining product quality. Although research [17,18,19] has examined the addition of fruits and vegetables in fish- and sauce-based products and their nutritional benefits, the specific incorporation of carrot pomace powder to enhance the antioxidant profile, flavor, and texture of these products remains underexplored. Food products are a source of nutrients and an alternative for culinary creativity and exploration. In many cultures, carp roe is a delicacy due to its unique texture and mild flavor. One such culinary creation is roe salad with the addition of carrot powder. This unique combination of delicate roe and nutritious carrot powder creates a harmonious blend of flavors, textures, and colors, offering a delightful culinary experience [20].

The present study aimed to investigate the functionality, chemical composition, and phytochemical characteristics of carrot pomace pulp. To achieve this objective, measurements of phytochemical and physicochemical composition, along with color, texture, rheological, and sensory analyses, were conducted to elucidate the potential applications of carrot pomace in the formulation of value-added carp roe salad. For this reason, the valorization of the by-products obtained during the industrial processing of carrots by using the carrot pulp exhausted in juice in the form of bioactive powder in the composition of the carp roe salad could offer numerous benefits to consumer health. By implementing strategies for by-product valorization, producers can reduce waste management costs, improve overall production efficiency, and create new revenue streams, contributing to a more sustainable and circular agri-food economy.

## 2. Materials and Methods

### 2.1. Chemicals and Reagents

Ethanol, Folin–Ciocâlteu’s reagent, ABTS (2,2-azino-bis(3-ethylbenzothiazoline-6-sulfonic acid) diammonium salt), catechine, sodium carbonate, aluminum chloride, sodium nitrite, sodium hydroxide, sodium acetate, acetone and hexane purchased from Sigma Aldrich (Millipore Sigma, Steinheim, Germany) was used for the analysis.

### 2.2. Obtaining Carrot Pomace Powder

A regional farmer in southeastern Romania provided fresh carrots (*Daucus carota* subsp. *sativus*, cv. Nantes). Carrots were peeled, rinsed, and subsequently processed for juice extraction utilizing a juicer (Philips HR1855, Amsterdam, The Netherlands), yielding the desired by-product, carrot pomace. The carrot pomace was then freeze-dried. Drying was carried out by lyophilization using a CHRIST Alpha 1-4 LD plus equipment, Germany, at −42 °C, under a pressure of 10 Pa, for 48 h. Finally, the dried pomace with a relative humidity of 10% was ground using an MC 12 kitchen grinder (Stephan GmbH, Berlin, Germany). The resulting powder was pulverized in a grinding mill for 50 s, with a mean particle diameter of 450 μm. Afterwards, the powder was collected, packed in polymer film, and stored at 20 °C until characterization and use.

### 2.3. Proximate Composition of Carrot Pomace Powder

The proximate composition of the carrot powder, including moisture, ash, protein, fat, fiber, and carbohydrate (by difference), was determined following the AOAC [21] standard procedures. All analyses were performed in triplicate, and results were expressed as g/100 g DW. Protein was determined using the Kjeldahl method (N × 5.7), lipid content by the Soxhlet extraction method, and ash content by incinerating 5 g of sample at 600 °C for 4 h in a muffle furnace, followed by cooling in a desiccator and reweighing. Moisture was measured by oven drying at 105 °C, and crude fiber was quantified using the enzymatic–gravimetric method described by Chantaro et al. [22]. Total carbohydrates were calculated by measuring the difference:

[100 − (moisture + protein + fat + ash + fiber) %].

### 2.4. Color Analysis of Carrot Pomace Powder

The color parameters of the carrot pomace powder were determined using an MINOLTA Chroma Meter CR-410 (Konica Minolta, Osaka, Japan), calibrated with a standard white plate in accordance with the manufacturer’s specifications. The measured parameters included L* (lightness), a* (red–green), and b* (yellow–blue) coordinates. Additionally, the hue angle [arctan(b*/a*)] and chroma [$\sqrt{{{(a}^{*})}^{2}+{{(b}^{*})}^{2}}$], representing visual color tone and intensity, respectively, were calculated.

### 2.5. Extraction of Biologically Active Compounds

To extract bioactive compounds from carrot powder, 1 g of carrot powder was homogenized with 9 mL solvent mixture (hexane: acetone 3:1) or 70% ethanol (only for extracting total polyphenols and flavonoids). Ultrasonically assisted solvent extraction was carried out; therefore, the mixture was maintained in an ultrasonic bath for 45 min at 30 °C, at an ultrasonic power of 200 W and 40 kHz. (Smart sonic cleaner MRC Scientific, Holon, Israel), followed by centrifugation at 6000 rpm and 4 °C for 10 min (Universal 320R cooled ultracentrifuge, Hettich, Tuttlingen, Germany) and the supernatant was collected [23].

Three successive extractions were performed to increase the extraction yield. Subsequently, the supernatants were gathered and concentrated to dryness at 40 °C under decreased pressure (AVC 2-18, Christ, Osterode, Germany). Characterization of concentrated extracts involved determining β-carotene concentration, total carotenoids, flavonoids, polyphenols, and antioxidant activity using solvent solubilization.

### 2.6. Carotenoid Content

The total carotenoid and β-carotene concentrations were assessed utilizing the methodology of Souza et al. [24]. Subsequently, 0.2 mL of the extract was diluted in a solvent mixture (hexane:acetone, 3:1) and then introduced into the UV quartz cuvette. Absorbance was quantified at two wavelengths: 450 nm for total carotenoids and 470 nm for β-carotene. The quantitative data were presented as the dry weight (DW) in milligrams per gram (mg/100 g).

The amount of total carontenoids and β-carotene was calculated according to Noshad et al. [13]:

(1) $$\mathrm{Total}\;\mathrm{carotenoids}\;(\mathrm{mg}/100\;g\;\mathrm{DW})\;=\frac{A\times Mw\times Df\times Vd}{m\times L\times Ma}$$

(2) $$\beta \text{-}\mathrm{carotene}\;(\mathrm{mg}/100\;g\;\mathrm{DW})=\frac{A\times Mw\times Df\times Vd}{m\times L\times Ma}$$

where A represents petroleum ether absorbance at the wavelength, Mw represents molecular weight (536.9), Df represents sample dilution rate, Vd the volume used, Ma represents molar absorptivity (2500 L·mol^−1^·cm^−1^ for carotenoids, and 2590 L·mol^−1^·cm^−1^ for β-carotene), m the weight used, and L represents spectrophotometer cell diameter (1 cm).

### 2.7. Determination of Total Phenolic Content

Initially, 500 μL of each sample extract was transferred into test tubes, followed by the addition of 2.5 mL Folin–Ciocâlteu reagent and 2 mL of 7.5% sodium carbonate solution. The tubes were then covered with aluminum foil and left to react at room temperature for 60 min. Subsequently, the absorbance was measured at 765 nm using a UV–Vis spectrophotometer (Biochrom Libra S22 UV/Vis, Cambridge, UK). The results were expressed as milligrams of gallic acid equivalent per gram of dry weight (mg GAE/g DW).

### 2.8. Determination of Total Flavonoid Content

Flavonoid contents were assessed using a modified aluminum chloride colorimetric method described by Pękal and Pyrzynska [25]. 250 µL of plant extract (1 mg/mL) was mixed with 1.25 mL of deionized water and 0.075 mL of 5% NaNO_2_ solution. 0.15 mL of 10% AlCl_3_ was added after 5 min in the dark. 0.5 mL of 1M NaOH and 0.775 mL of deionized water were added to the reaction mixture after 6 min. The absorbance was red at λ = 510 nm using a UV-Vis spectrophotometer (Libra S22, Biochrom, Cambridge, UK). Catechin was used as a standard, and the flavonoid content was expressed as catechin equivalent per gram of dry weight (mg CE/g DW).

### 2.9. Determination of the Antioxidant Activity

The antioxidant activity of carrot powder was determined using the ABTS (2,2′-azinobis (3-ethylbenzothiazoline-6-sulfonic acid) diammonium salt) radical scavenging method [26]. To achieve this, 0.02 mL of extract and 1.98 mL of ABTS solution were prepared and subsequently allowed to remain at room temperature for 2 h, protected from light. The Libra 22 UV/Visible spectrophotometer was employed to quantify the mixture’s decrease in absorbance at 734 nm. The results were expressed in terms of inhibition percentage and µM Trolox Equivalent/g dry weight (DW) (µM TE/g DW).

### 2.10. Determination of the Carotenoids by HPLC

Chromatographic analysis was used to characterize carotenoids, according to Gavril et al. [27]. A Finnigan Surveyor HPLC system with a DAD UV–visible detector (Thermo Scientific, Waltham, MA, USA) was used to acquire the extract’s chromatographic profile. The Xcalibur 2.0.7 software controlled the equipment. Carrot extract carotenoid components were analyzed at 450 nm using a Lichrosorb RP-18 (5 μm) Hibar RT 125–4 column. The mobile phase comprised two solvents: 90% acetonitrile (A) and 100% ethyl acetate (B). The injection volume was 10 μL, and the flow rate was 1.0 mL/min. The elution gradient consisted of the following intervals: 0–16 min at 15% B; 16–54 min transitioning from 15% to 62% B; 54–56 min at 62% B; 56–60 min transitioning from 62% to 15% B; and 60–70 min at 15% B. The primary carotenoids were quantified using calibration curves from known standards and compared with reference chromatographic profiles and retention time data available in scientific literature.

### 2.11. Carp Roe Salad Preparation

To produce the enhanced product and evaluate its functionality, two distinct ratios of carrot powder were utilized (6%-I1 and 12%-I2). These concentrations were selected based on previous research and nutritional equilibrium, with the goal of enhancing both the sensory and functional properties of the resulting product. The concentrations of carrot powder at 6% and 12% were selected to investigate their effects on the physicochemical properties and sensory attributes of the obtained product.

Value-added carp roe contains the following ingredients, % (*w*/*w*): carp roe (90 g), sunflower oil (300 g), lemon juice (25 g), water (50 g), and carrot powder (I1–6% (27.9 g) and I2–12% (55.8 g)). The procedure for obtaining roe is low-complexity technique and involves mixing the ingredients presented above, adding carrot powder, and relating the amount of roe. For comparison, a control sample was also prepared, which followed the same technology but without the addition of carrot powder. The sequence of stages is as follows: The ingredients in the recipe were weighed. The carp roe (cleaned and lightly salted) was placed in a bowl and homogenized for 1–2 min until a whitish emulsion was formed.

Sunflower oil was gradually added with each installment, in amounts of one to two tablespoons. The mixture was continuously homogenized for 10–15 min, and once the roe salad began to lighten in color, the sunflower oil was alternated with lemon juice until fully homogenized. When the salad’s consistency became too dense, water was added gradually and homogenized until the salad became compact and fluffy.

Carrot powder (at proportions of 6% and 12% *w/w*) was then added and homogenized until completely dissolved into the product mass. The samples were packed into glass jars (150 g) and stored at 0–4 °C for up to 14 days to allow for physicochemical and phytochemical analyses.

### 2.12. Proximate and Phytochemical Composition

The roe with added value was characterized from a physicochemical point of view, using AOAC [28] guidelines.

The proximate and phytochemical composition, including moisture, crude protein, crude fiber, lipids, carbohydrates, and ash content in carrot powder and carp roe salad, was analyzed according to AOAC [28] guidelines. The same methods were employed to assess the overall carotenoid, phenolic, flavonoid, and antioxidant activities in the carp roe salad enriched with carrot powder.

### 2.13. Color Analysis

Carp roe salad samples were analyzed for CIELAB colorimetric parameters using a portable colorimeter with illuminator C (Chroma Meter, model CR-410, Konica Minolta, Osaka, Japan), which was standardized using a white reference tile before each measurement. The results were expressed as L* (brightness), a* (tendency to red for an a* “+” or green for an a* “−”), and b* (tendency to yellow for b* “+” or blue for b* “−”). The Chroma, hue angle, and total color difference (ΔE) were calculated using the formula provided by Nistor et al. [29].

### 2.14. Texture Analysis

The textural properties of value-added roe were evaluated using the Texture Profile Analysis (TPA) method applied with a Brookfield texture analyzer (model CT3-1000, AMETEK Inc., New Bedford, MA, USA). This consisted of a double penetration of a test device to a depth of 15 mm inside the sample packed in a cylindrical plastic container, having a height of 30 mm and a diameter of 43 mm.

The penetration speed was set at 1 mm/s, with a sensitivity threshold of 0.067 N. The textural parameters evaluated included firmness (N), defined as the maximum resistance exerted by the sample during the first penetration cycle; adhesion (mJ), defined as the energy required to detach the sample from the testing probe; cohesiveness (dimensionless), representing the strength of internal bonds that provide structural consistency; and elasticity (mm), describing the ability of the product to recover from deformation during testing. Data acquisition and processing were performed using TexturePro CT V1.5 software.

### 2.15. Rheological Analysis

Dynamic oscillatory and stepped flow tests were carried out with an AR2000ex rheometer (TA Instruments, Ltd., New Castle, DE, USA). A 40 mm 2o steel cone geometry with a gap of 2000 mm and a Peltier plate temperature control system was used for the rheological testing. The registered results were analyzed through TA Rheology Advantage Data Analysis Software V 4.8.3. (TA Instruments, New Castle, DE, USA).

Each sample’s linear viscoelastic region (LVR) was first identified by running strain sweep tests at increasing oscillating strain in the 0.1–100% domain and at a constant frequency of 1 Hz. Further frequency sweeps were performed in the LVR to check the small-amplitude oscillatory shear behavior of the samples. The roe samples’ elastic modulus (G′) and viscous modulus (G″) were measured as a function of oscillating frequency in the 0.1–100 Hz domain.

The samples’ apparent viscosity (η, Pa∙s) was measured while the shear rate (γ, s^−1^) increased from 0.1 to 100 s^−1^ over the steady shear test. All rheological measurements were carried out at 20 °C.

### 2.16. Sensorial Analysis

The sensorial properties of the carp roe salad samples were analyzed by 20 panelists from the “Ion Ionescu de la Brad” Iasi University of Life Sciences. Twenty untrained panelists aged 18–39 years, consisting of 40% males and 60% females, who reported consuming fish products more than once per month, were randomly selected for the sensory evaluation.

Appearance, odor, consistency, color, taste, aroma, aftertaste, firmness, cohesiveness, and acceptability were evaluated on a nine-point scale (1—extremely dislike; 9—extremely like) [30]. The samples were presented to the panelists in transparent, colorless plastic containers, each labeled with a unique three-digit identification code. To neutralize residual flavors between evaluations, bread and drinking water were provided for palate cleansing.

### 2.17. Data Analysis

The data represented the mean of triplicate analyses ± standard deviation. The assessed parameters performed one-way ANOVA to ascertain the mean differences among the extracts. Upon detecting significant differences in ANOVA (*p* < 0.05), Tukey’s multiple range test was employed at *p* < 0.05, utilizing the SPSS Statistical program (SPSS version 20, IBM Inc., Armonk, New York, NY, USA).

## 3. Results and Discussions

### 3.1. Global Characterisation of Orange Carrot Powder

Table 1 presents the phytochemical composition, color, and nutritional characteristics of the extract derived from carrot powder.

Multiple studies examining carrot pomace indicated favorable outcomes concerning the antioxidant activity of this botanical material [31,32]. Following these tests, we achieved a notable result of 1182.45 ± 4.64 µM TE/g DW for the extract. The phytochemical evaluation of the extract revealed significant antioxidant activity, characterized by greater amounts of carotenoids and polyphenols, as illustrated in Table 1. A significant polyphenol concentration of 138.11 ± 0.94 mg GAE/100 g DW was recorded for carrot extract. In addition to polyphenol concentration, carotenoids augment the antioxidant efficacy of carrot extract. The analysis of the carotenoids of carrot extract indicated a total carotenoid concentration of 289.10 ± 0.45 mg/100 g DW.

The current results regarding the bioactive properties of carrot powders were notably higher than those previously reported by other studies [33,34]. The carotenoid content in carrot pomace ranges between 78.66 and 120.14 mg/100g, as reported by Borowska et al. [33]. According to Hernandez-Ortega et al. [34], carrot pomace has 65.74 to 92.64 mg/100 g of total carotenoid content, with β-carotene levels ranging from 6.83 to 15.81 mg/100 g. Phenolics and other bioactives are influenced by various factors, including cultivar, harvest time, growing conditions, and the type of solvent used for extraction [35].

Bouzari et al. [36] also studied the total polyphenols in fresh carrots. According to the findings, raw carrots had a total polyphenol content of 108 mg GAE/100 g dry matter, lower than ours. The content of total polyphenols improved relatively after 24 h of freezing, reaching levels of about 133 mg GAE/100 g dry matter. Borowska et al. [33] analyzed fresh, convective, dried, and lyophilized carrot pomace, finding better phenolic extractability in dried materials and showed a significant increase in phenolic content by approximately 2.5% for convective dried carrot pomace and around 20% for lyophilized carrot pomace using ultrasound-assisted extraction of 80% methanol (*v*/*v*) and 20% water (*v*/*v*). The maximum concentration was observed in lyophilized pomace (1188.04 mg GAE/100 g DW), followed by convective dried pomace (1013.02 mg GAE/100 g DW) and fresh pomace (987.88 mg GAE/100 g DW). Additionally, the ability to scavenge DPPH radicals was higher in the dried pomace. Hernandez-Ortega et al. [34] reported higher total phenolic contents in carrot pomace—whether fresh, convectively dried, or microwave-dried—likely influenced by the carrot variety rich in phenolics and variations in juice extraction or drying processes. The authors recorded 1841 mg gallic acid equivalents (GAE) per 100 g dry matter (DM) for fresh pomace, 1505 mg GAE/100 g DM for convectively dried pomace, and 1412 mg GAE/100 g DM for microwave-dried pomace, using 80% acetone as the extraction solvent. The content of phenolic and bioactive substances was different because it varies depending on the cultivar, time of harvest, growing conditions, and extraction solvent.

The carrot pomace powder had a moisture content of 10.01 ± 0.14 g/100 g DW, with dry ash, protein, carbohydrate, fat, and fiber contents of 9.79 ± 0.09, 9.82 ± 0.11, 26.45 ± 0.23, 0.27 ± 0.04, and 42.93 ± 0.24 g/100 g DW, respectively. Carrot pomace includes 4–5% protein, 8–9% reducing sugars, 5–6% minerals, and 37–48% dietary fiber on a dry weight basis [37]. Luca et al. [38] reported that for the Sirkana variety of carrot pomace, the protein content was 9.14 ± 0.06%, fat content was 1.13 ± 0.03%, ash content was 5.89 ± 0.01%, fiber content was 31.40 ± 0.70%, moisture content was 5.91 ± 0.15%, and carbohydrates accounted for 46.55 ± 0.64%.

The color characteristics of carrot extract revealed a lightness of 70.95 ± 0.49, with a positive a* value of 10.02 ± 0.18, indicating red hues, and a positive b* value of 29.62 ± 0.25, indicating yellow hues. Based on the color indices, the powder was found to be positioned in the first quadrant (+a*, +b*). The color parameters of carrot pomace varied according to variety, especially in luminosity (L*), red nuance (a*), and yellow nuance (b*). Adeleye et al. [39] reported that in dried carrot pomace, the L* value was 48.52 ± 0.76, a* was 16.56 ± 0.39, b* was 22.34 ± 0.66, and the hue was 0.48 ± 0.03. Luca et al. [38] reported that for the Sirkana variety of carrot pomace, the L* value was 69.44 ± 0.01, a* was 9.25 ± 0.06, and b* was 19.56 ± 0.04. The differences between our results and other research may be attributed to the components of the matrix and the wide variety of carrots.

The results above underscore carrot powder’s potential as a natural food ingredient. In conclusion, the valorization of carrot juice by-products offers a promising economic pathway toward sustainable food production, enabling cost reduction, resource efficiency, and the development of high-value functional products that support a circular agri-food economy.

### 3.2. Determination of the Carotenoids by HPLC

**Figure 1:**
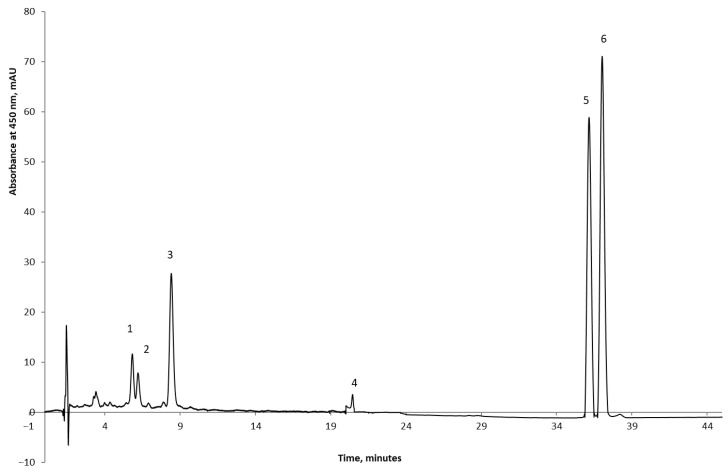
The HPLC profile of the carrot powder: 1—lutein; 2—zeaxanthin; 3—not identified; 4—not identified; 5—α-carotene; 6—β-carotene.

The chromatogram in Figure 1 illustrates a high-performance liquid chromatography (HPLC) separation of carotenoid compounds derived from carrot pomace powder. Detection occurred at 450 nm, optimal for carotenoids according to their high absorption in the visible spectrum. The carotenoid compounds identified were lutein (1), zeaxanthin (2), α-carotene (5), and β-carotene (6). The prominent peaks at retention durations of about 36 and 37 min correspond to α-carotene and β-carotene, respectively, aligning with research indicating they are the primary carotenoids in carrot roots [40,41,42]. Lutein and zeaxanthin, eluting earlier because of their higher polarity, are found in reduced amounts. Similar results were obtained by Purkiewicz et al. [41] established that orange carrot juice mostly contained α- and β-carotene and minor amounts of lutein, zeaxanthin, and 13-cis-β-carotene, exhibiting variability according to cultivar.

Zelenkova et al. [43] developed an HPLC-DAD technique utilizing a C18 column and an isocratic mobile phase of ACN–MeOH–ethyl acetate to assess 11 carrot varieties. Principal carotenoids identified were β-carotene (55–68%), α-carotene (30–40%), and lutein (1–6%) of the overall composition. Nunez-Gomez et al. [44] used a C30 column and separated lutein (~10.5 min), β-cryptoxanthin (~14.3 min), and β-carotene (~17.9 min).

### 3.3. Physicochemical Characterization of Value-Added Carp Roe Salad Samples

The value-added carp roe salad was analyzed from a physicochemical perspective, as presented in Table 2. Comparing the chemical content of the carp roe salad to the control, the addition of carrot powder significantly improved it.

Table 2 indicates that the protein and fat contents of the carp roe salad samples including carrot powder are inferior to those of traditional roe salad. Additionally, the moisture content of the carp roe salad is reduced by the addition of carrot powder. The decrease in protein and lipid contents observed in sample I2 compared to I1 can be attributed to a dilution effect, as the higher proportion of carrot powder—naturally lower in these macronutrients—replaces part of the roe matrix, thereby reducing their relative concentration in the final product, consistent with findings by Bekele et al. [45].

From the perspective of carbohydrate content, an increase is observed with the percentage of added powder. Carbohydrates rose to 5.3 g/100 g in I1 and 8.7 g/100 g in I2. Compared to the control sample, the carp roe salad samples, which include 12% carrot powder, show an improved nutritional value. This addition significantly contributes to the total amount of insoluble fiber in the product. Specifically, insoluble fibers increased from 0 g/100 g in the control to 2.6 g/100 g in I1 and 4.09 g/100 g in I2.

Therefore, the beneficial effects of carrot powder on the nutritional quality of carp roe salad are largely attributed to its high fiber level. Although the energy value of the product with the added powders is nearly similar to that of the conventional product. These compositional shifts reflect the contribution of carrot powder as a source of dietary fiber and carbohydrates.

Using vegetable powders enables the production of nutritious snacks, as the vegetable components are nutritionally enhanced. Ying et al. [46] reported a fiber content of 4.7 g/100 g in snacks with a 20% carrot powder addition and 23.7 g/100 g fiber in snacks with a 100% addition of carrot powder. The amount of carbohydrates expressed in g/100 g reported for these carrot-based snacks was 56 and 78, respectively, for the same percentage of added powder.

### 3.4. Phytochemical Characterization and Antioxidant Potential Evaluation of Value-Added Carp Roe Salad Samples

The ABTS free radical assay measured antioxidant activity, and phytochemical analysis highlighted the significance of the roe salad samples. Table 3 shows the results.

Increasing the carrot powder supplementation from 6% to 12% resulted in significant improvements in carotenoid, flavonoid, and polyphenol levels in the enriched carp roe salad samples compared to the control. Powder incorporation also improved the ABTS scavenging capacity of the carp roe salad, with a significant increase, particularly in the I2 sample. All carp roe salad samples exhibited significant differences (*p* < 0.05) in antioxidant activity and phytochemical characteristics. The incorporation of carrot pomace at concentrations of 6% and 12% into the formulation of carp roe salad resulted in a significant enhancement of its nutritional and functional properties. Specifically, the levels of bioactive compounds showed marked increases: total carotenoids rose to values between 84.01 and 111.01 mg/100 g DW, flavonoid content increased to 21.85–28.72 mg CE/100 g DW, and total polyphenols reached levels ranging from 66.18 to 88.91 mg GAE/100 g DW. In parallel, the antioxidant capacity of the product was significantly improved, with values increasing to 528.16–588.32 μM TE/g DW. These results indicate that the addition of carrot pomace not only enriches the salad with health-promoting compounds but also contributes to its overall antioxidant potential, thereby enhancing its functional food value.

Similarly, Hernandez-Ortega et al. [34] incorporated microwave or hot-dried carrot pomace powders into cookies to enhance their phytochemical content. By replacing 30% of wheat flour with either carrot pomace powders, the cookies showed a 3.7-fold increase in total dietary fiber, contributing 7.4% of the daily fiber intake with one cookie. A similar increase was observed in carotenoids and phenolic compounds. Cookies with microwave-dried carrot pomace powder contained the highest levels of β-carotene, gallic acid, and ferulic acid. The resulting cookies had an appealing orange color, improved carotenoid content, and better antioxidant and sensory characteristics, as confirmed by other studies [47,48,49].

The results presented in Table 3 confirm the added value of roe with the addition of carrot powder, as it increases the total content of carotenoids and polyphenols, resulting in a product with higher antioxidant activity compared to the control sample. Thus, incorporating the powder as a source of carotenoids in the carp roe salad samples improved their nutritional and functional composition.

### 3.5. Colorimetric Parameters of Value-Added Crap Roe Salad Samples

Considering that the color parameter of crap roe salad significantly influences customer acceptance, it is essential to assess it comprehensively [50]. Roe salad samples were analyzed for CIELAB colorimetric parameters, and the results were expressed as L*, a*, b*, chroma, hue angle, and ΔE presented in Table 4. The results in Table 4 indicate that the value-added carp roe salad samples (I1 and I2) exhibit varying colors of yellow. The color intensity is directly proportional to the quantity of carrot powder incorporated.

The carp roe salad samples presented the highest values of yellowness and redness and the lowest values of lightness.

The carp roe salad samples obtained with the addition of carrot powder showed a yellowish-orange color; the shade of the samples varies with the percentage of added powder (b* was 10.51 in C, 15.57 in I1, and 22.01 in I2). This characteristic demonstrates that carrot powder possesses a high coloration capacity due to the carotenoid pigments and can be employed to improve the appearance of roe salad, thereby increasing the product’s appeal to consumers. Thus, incorporating carotene-rich carrot pulp powder gave the value-added carp roe salad samples a more attractive color. Similarly, in the study by Pathare [50], which used carrot powder as a plant material to produce value-added biscuits, the color analysis showed that varying amounts of carrot powder significantly influenced the biscuits’ color. The L* values decreased significantly (*p* < 0.001), while b* values increased with higher levels of carrot powder in all the biscuit samples, reflecting the characteristic orange hue of the carrot powder.

Kultys and Moczkowska-Wyrwisz [51] demonstrated that adding carrot juice to pasta resulted in the following color characteristics: the control pasta exhibited a uniform light yellow color without discoloration. The Lab* values for fresh pasta were L* 65.78 ± 1.93, a* 0.48 ± 0.22, and b* 22.65 ± 2.86, which aligned with the findings reported by Gull et al. [52] and Piwińska et al. [53].

The hue angle value measures the chromaticity or tone of the color, while the chroma value represents color saturation. The Chroma values varied between the control and the enhanced samples, showing similarities in the b* color coordinate (Chroma was 11.04 in C, 16.07 in I1, and 22.52 in I2). The color tone values ranged from 1.26 to 1.36, with an increased trend with powder addition. The overall color shifts in the samples ranged from 9.75 to 16.88 units, influenced by the addition of carrot powder. Increasing the carrot powder content led to higher ΔE values. The natural hue of the carrot powder is a clear indication of the effect it has on the color of the carp roe salad samples.

### 3.6. Texture Analysis of Value-Added Crap Roe Salad Samples

The texture of the sauce is a critical component of the overall evaluation of the grade of the product. For consumers, it is one of the most important properties of sauce that they pay attention to, according Gull et al. [52]. The Texture Profile Analysis (TPA) test aims to imitate the chewing of food by human jaws. It provides objective information regarding hardness, adhesiveness, cohesion, elasticity, gumminess, or chewiness. The texture parameters are presented in Table 5.

The findings show that the presence of carrot pomace significantly affected the textural properties of the carp roe salad samples. Furthermore, the carp roe salad with the highest carrot pomace content exhibited the most pronounced alterations in its textural characteristics when contrasted with the control carp roe salad. The carp roe salad samples exhibited a decrease in cohesiveness (from 0.11 to 0.09 and 0.07) and elasticity (from 3.88 to 3.70 and 2.71) and an increase in hardness (from 0.21 to 0.38 and 0.52) and adhesiveness (from 2.48 to 3.99 and 4.25) as a result of the carrot pomace’s incorporation. As stated by Chang et al. [54], an important component to consider is the firmness of roe salad samples, as it determines both the sensory traits (mouthfeel) and the applicability of the product. According to the findings detailed in Table 5, it can be inferred that the incorporation of carrot powder led to higher firmness levels in samples I1 and I2 compared to the control sample. This characteristic results from compounds in the plant material that play a stabilizing role, such as pectin [55].

The texture profile analysis of bread conducted by Mildner-Szkudlarz et al. [56] demonstrated that the hardness of bread is considerably increased (*p* < 0.05) by the assimilation of carrot pomace powder. The dilution of gluten as a result of the addition of carrot pomace powder may be the cause of the harder texture.

In addition, incorporating carrot powder into the carp roe salad recipe helped improve stickiness, resulting in a fine, fluffy texture to the finished product. Cohesiveness measures the sample’s resistance to a second deformation [57], defined as the strength of the internal bonds between the constituent ingredients of roe salad. For the control sample, the cohesiveness was 0.11, but it decreased to 0.09 and 0.07, respectively, with the increasing percentage of carrot powder. Thus, an inversely proportional variation in cohesiveness with firmness and added powder concentration can be observed, which may indicate a disintegrated development capacity during mastication. The ability of the roe samples to recover from the deformation of the first penetration cycle represents their elasticity [56]. Thus, the samples’ elasticity decreases with the increase in the number of added ingredients, which is associated with a solid behavior of the samples.

Kultys and Moczkowska-Wyrwisz [51] showed that the hardness of the paste was similar across all the tested products and did not exceed a force of 2 N. However, a clear relationship between the amount of expeller and the product’s hardness was observed. As the product’s proportion of fruit and vegetable pomace increased, its hardness increased significantly. In a double compression test, the highest resistance was recorded during the first compression for paste with 10% of the additive, with a value of 1.78 ± 0.24 N for carrot pomace. Overall, the carrot powder improved the texture properties of the obtained products.

### 3.7. Rheological Properties of the Carp Roe Salad

The carp roe salad samples supplemented with various levels of carrot powder were also characterized regarding rheological properties by performing stepped flow and low oscillatory tests.

**Figure 2:**
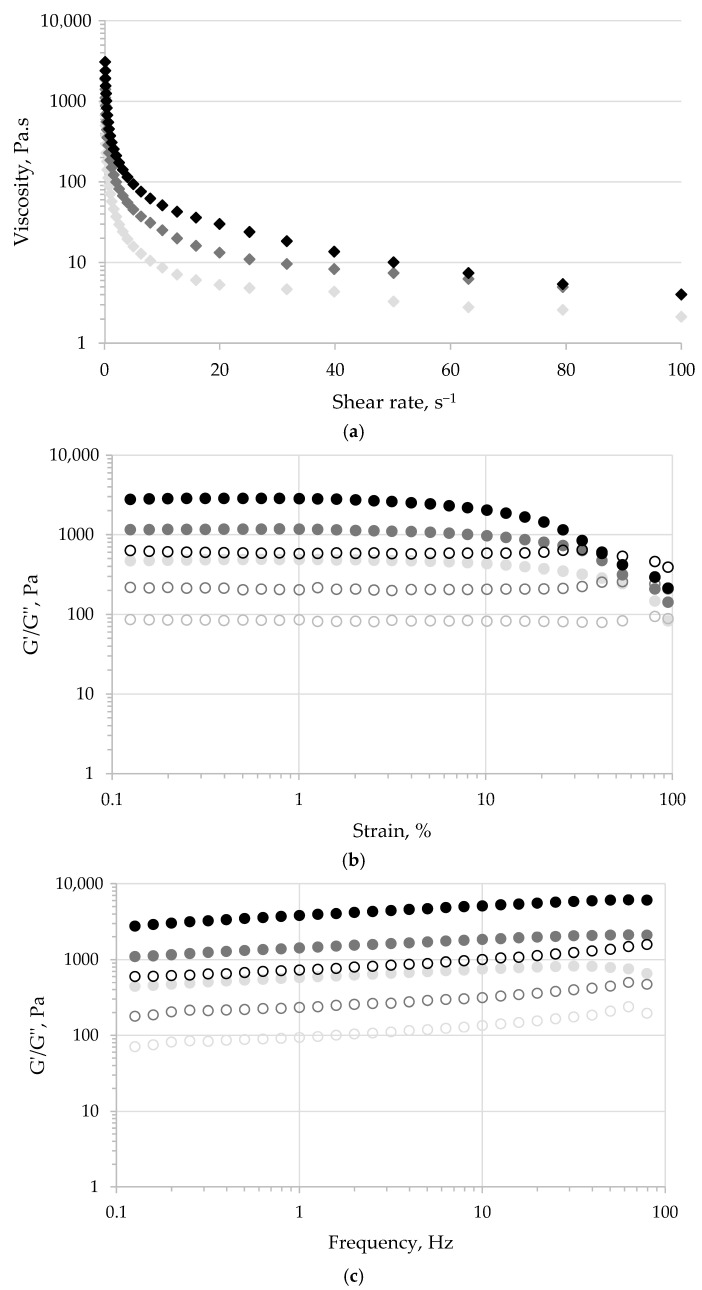
Rheological behavior of the carp roe salad with no (light gray), 6% (dark gray), and 12% (black) carrot powder over the stepped flow (**a**), strain sweep (**b**), and frequency sweep (**c**) tests. G′ and G″ are represented with full and open circles, whereas viscosity values are represented with diamonds.

The steady shear flow behavior is presented in Figure 2a. Regardless of the carrot powder addition, all samples exhibited shear-thinning behavior specific to pseudoplastic materials. As expected, the rise in the carp roe salad dry weight due to the addition of carrot powder caused a significant increase in the samples’ viscosity (Table 6). The apparent viscosity values decreased over the entire tested shear rate domain (Figure 2a, Table 6), indicating the gradual weakening of the molecular interactions due to increasing shearing. The apparent viscosity decrease was more pronounced in the first part of the shear rate domain, most probably due to the breaking of the intermolecular hydrogen bonds [58]. The interfacial forces contributing to the emulsion droplets’ stability appeared to be overcome by the hydrodynamic forces developed at high shear rates, causing the gradual disruption of the droplet aggregates and potential flocculation [59,60]. Regardless of the carrot powder level, a plateau of apparent viscosity values was reached at a shear rate higher than 50 s^−1^, illustrating similar flow behavior of the analyzed samples during processing through pumping [58].

The LVR of the carp roe salad samples registered over the strain sweeps are presented in Figure 2b. Outside the LVR, the strain value corresponding to the G′-G″ cross-over decreased from 94.7% to 46.3% with the carrot powder addition level. These critical strain values, corresponding to the phase inversion, measure the maximum deformation that can be applied to the samples without permanently altering the specific structure of carp roe salad [58]. The results presented in Figure 2b suggest that carrot powder addition influenced the yield point and the start of the flow. Consequently, the roe salad sample’s stability decreased in conjunction with the increase in viscosity caused by the addition of carrot powder at higher concentrations.

Figure 2c illustrates the findings of the frequency sweep experiments conducted within the LVR. Regardless of the carrot powder level, all roe salad samples exhibited significantly higher G′ values than G″ with no cross-over point, and both dynamic moduli increased over the whole frequency domain (Figure 2c). These results suggest that all samples presented elastic solid-like behavior, specific to the structured food systems with permanent interaction between molecules and aggregates [58,61,62].

### 3.8. Sensory Analysis of Value-Added Crap Roe Salad Samples

**Figure 3:**
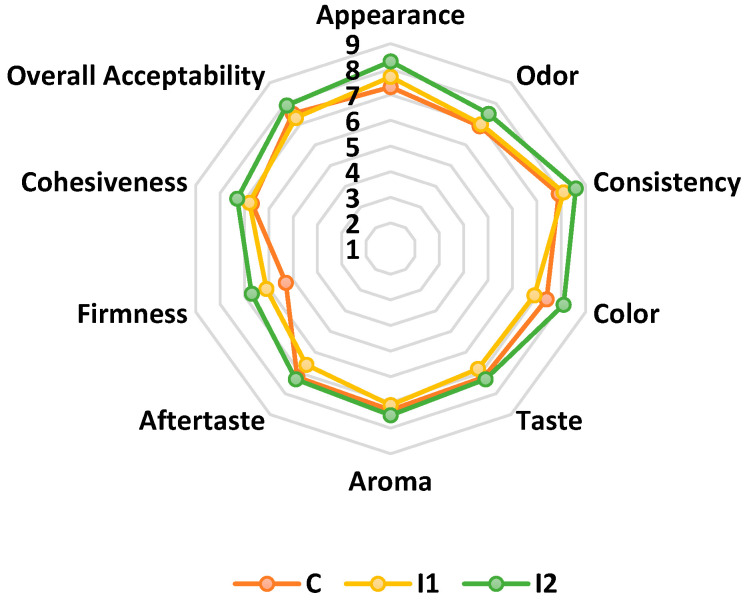
Comparative diagram of sensory attributes specific to the types of carp roe salad: C—carp roe salad without adding carrot powder, I1, and I2—carp roe salad with 6 and 12% carrot powder.

From a sensory point of view, the carp roe salad samples were analyzed using the following 10 attributes: Appearance, Smell, Consistency, Color, Taste, Aroma, Aftertaste, Firmness, Cohesiveness, and General acceptability using the scoring scale method from 1 to 9. The results are shown in Figure 3.

The appearance of carp roe salad is presented as a homogeneous emulsion obtained from carp roe of the appropriate size for the species and sunflower oil. The consistency of the carp roe salad is compact, bound, and specific to the product without separate oil. Both salads are creamy and smooth, with a soft texture that is easy to spread. The enriched salads have a more vibrant orange color with a smooth and creamy texture.

The product’s smell and taste are characteristic, with a distinct aroma and no foreign flavors or odors, such as rancidity or excessive oil. The aroma of the sample salads is mild, with a subtle earthy scent from the carrot powder.

The assessment of the sensory evaluation of value-added crap roe salad indicates that the variants incorporating carrot powder were characterized as possessing an appealing and agreeable taste, aroma, and color, aligning with the standard product, and exhibiting a compact consistency.

**Figure 4:**
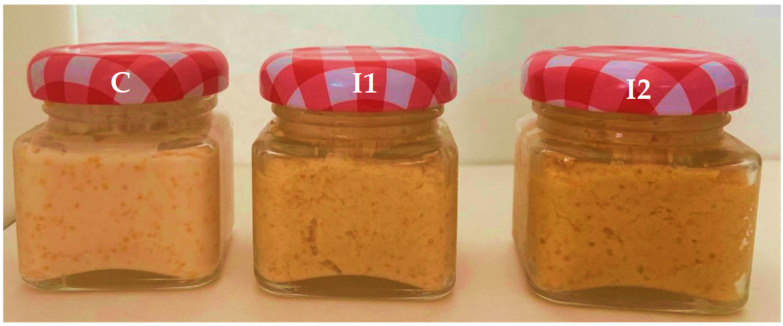
Carp roe salad with different percentages of carrot powder: C—carp roe salad without adding carrot powder, I1, and I2—carp roe salad with 6 and 12% carrot powder.

The greatest color score was achieved for the roe salad with the use of 12% powder (I2), emphasizing its more vivid and appealing dark yellow tone (Figure 4). The color of unprocessed roe samples is pink-yellowish for carp roe. Sensory analysis of the crap roe salad showed that the addition of carrot powder did not affect the acceptability of the crap roe salad as perceived by the panelists. The resulting carp roe salad presented a consistency specific to the traditional product, a pleasant yellow to orange color, typical of carrots, a pleasant taste, and a homogeneous texture, which combines the aroma of lemon with that of carrot, representing a source of carotenoids.

**Figure 5:**
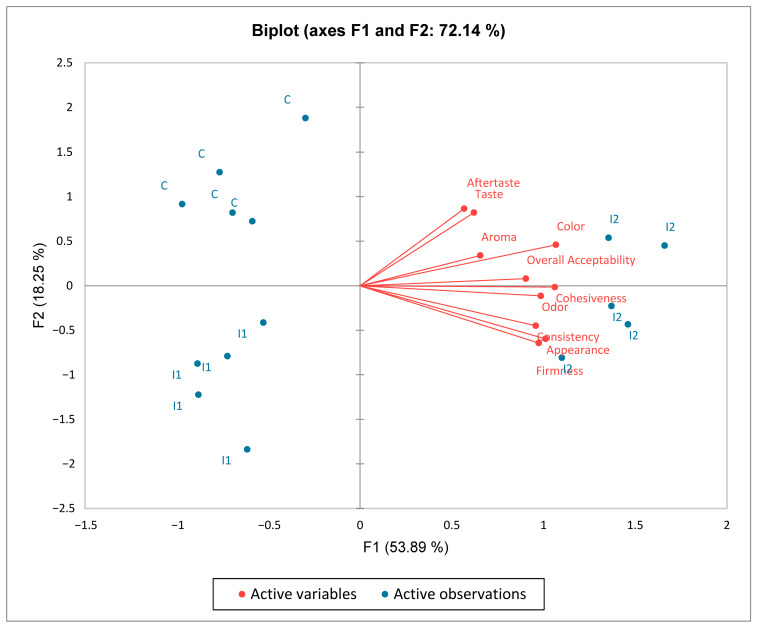
Principal component analysis biplot of the position of the three carp roe salads (C, I1, and I2) for sensory attribute evaluation.

The biplot of the Principal Component Analysis (PCA) (Figure 5) visualizes the changes and relationships among the evaluated sensory qualities and the carp roe salad batches. The attributes in the upper-right quadrant, namely aftertaste, taste, aroma, color, and overall acceptability, were positively positioned in the first axis (F1), signifying a positive connection. Furthermore, appearance, odor, consistency, firmness, and cohesiveness positively influence axis F1. The placement of these sensory attributes suggests that I2, situated nearest to these characteristics and on the positive side of the biplot, is favorably regarded by consumers concerning appearance and overall evaluation. The two axes accounted for 72.14% of the total variation. An analysis of the PCA results indicates that I2 has emerged as the leader in consumer preferences. It was distinguished by a noticeable carrot flavor, imparting a slightly sweeter and more savory note to the salad while still maintaining a harmonious balance with the roe’s salty taste.

The analysis of the sensory evaluation results for the value-added carp roe salad samples indicates that the I2 variant was the most favored by the panel of tasters, exhibiting a balanced and pleasant taste, aroma, and fragrance, along with a compact, fine consistency, and a powder that enhances the color attributes and overall acceptability of the products. Consequently, carrot powder is a natural colorant and an appropriate ingredient for the production of crap roe salad, which has enhanced antioxidant and functional properties. The panelists have expressed their satisfaction with the resulting products.

In other studies, Gölge et al. [30] found that cakes with 3% carrot pomace were preferred over those with 5% and 7% pomace in appearance and texture. Cakes with 3% pomace were rated higher in texture than those with 5% or 7%. Additionally, no significant difference was found in the overall impression between cakes with 3% and 5% pomace, while cakes with 7% pomace were rated lower. According to Kultys and Moczkowska-Wyrwisz [51], the highest evaluation in the consumer acceptance tests was obtained for pasta with 10% carrot pomace, comparable to the evaluation of the control pasta. Mishra and Bhatt [63] evaluated the physical properties, chemical composition, texture, and sensory qualities of fiber-enriched pasta that they made by replacing refined wheat flour with carrot pomace powder at concentrations of 5%, 15%, and 25%. However, the texture scores decreased as the supplementation level increased, despite the fact that carrot pomace powder improved the pasta with an appealing color. Pasta with 15% carrot pomace powder was the most acceptable due to its appealing appearance, better taste, and flavor. Kumar et al. [64] incorporated carrot pomace as a dietary fiber source into wheat flour-based cookies, adding dried carrot pomace in proportions ranging from 0% to 9%. With the rise in carrot pomace proportion, moisture content, hardness, and L* (lightness) and a* (red vs. green) values also rose, however no significant alteration was noted in the b* (yellow vs. blue) value. Sensory scores for all cookies ranged from fair to very good, with the cookies containing 6% carrot pomace receiving the highest score.

## 4. Conclusions

The current study identified carrot pomace as a substantial source of fibers and carotenoids, underscoring its potential to enhance the nutritional quality of food products in which it is included. Adding carrot pomace to the carp roe salad enhanced its nutritional value, particularly by increasing its fiber and carbohydrate content. At the same time, the study indicates that using carrot powder in carp roe salad increases the level of bioactive compounds, especially carotenoids, and the antioxidant potential of the obtained product. The rheological response of the carp roe salad samples depended on the level of carrot powder addition. The inclusion of carrot powder raised the samples’ apparent viscosity while decreasing their structural stability. Additionally, carrot powder enhances the sensory and textural qualities of the final product. Adding carrot powder to the carp roe salad brings a unique color and texture to the dish, complementing the carp roe’s natural flavors. The deep orange color of the carrot powder enhances the visual appeal of the carp roe salad. Consequently, the carrot powder-based carp roe salad serves as evidence of culinary innovation and creativity. The results obtained in the study support the multifunctionality of the powder obtained from carrot pulp exhausted in the juice, which is a key component of the carp roe salad, as an important source of natural compounds with antioxidant, coloring, and flavoring activities.

For these reasons, the valorization of the by-products (carrot pulp exhausted in juice) obtained during the industrial processing of carrots by using them as a source of biologically active compounds represents a viable alternative to the variants of synthetic dyes, flavorings, and antioxidants. It can have multiple destinations, such as the food, nutraceutical, and pharmaceutical industries.

## Figures and Tables

**Table 1:** Comprehensive analysis of the carrot powder extract.

Characteristics	Extract from Carrot Powder
Total carotenoids, mg/100 g DW	289.10 ± 0.45
β-Carotene, mg/100 g DW	59.22 ± 0.29
Total flavonoids, mg CE/100 g DW	129.09 ± 0.78
Total polyphenols, mg GAE/100 g DW	138.11 ± 0.94
Antioxidant activity, µM TE/g DW	1182.45 ± 4.64
Ash, g/100 g DW	9.79 ± 0.09
Moisture, g/100 g DW	10.01 ± 0.14
Crude protein, g/100 g DW	9.82 ± 0.11
Crude fat, g/100 g DW	0.27 ± 0.04
Carbohydrates, g/100 g DW	26.45 ± 0.23
Total dietary fiber, g/100 g DW	42.93 ± 0.24
L*	70.95 ± 0.49
a*	10.02 ± 0.18
b*	29.62 ± 0.25
Hue angle	1.24 ± 0.05
Chroma	31.26 ± 0.29

DW—dry weight.

**Table 2:** Physicochemical characteristics of value-added roe salad samples.

Physico-Chemical Characteristics	Samples of Carp Roe Salad		
	C	I1	I2
Moisture, g/100 g	27.5 ± 1.38 ^a^	26.4 ± 0.05 ^b^	25.1 ± 0.11 ^c^
Protein, g/100 g	5.4 ± 0.09 ^a^	5.1 ± 0.03 ^b^	4.6 ± 0.06 ^c^
Lipid, g/100 g	65.2 ± 2.20 ^a^	63.2 ± 3.12 ^b^	61.6 ± 5.04 ^c^
Carbohydrates, g/100 g	0 ^c^	5.3 ± 0.60 ^a^	8.7 ± 0.43 ^b^
Insoluble fibers, g/100 g	0 ^c^	2.6 ± 0.02 ^a^	4.09 ± 0.04 ^b^
Energetic value:			
kcal	628.5 ± 0.11 ^b^	630.4 ± 0.12 ^a^	627.41 ± 0.10 ^c^
kJ	2629.64 ± 0.11 ^b^	2637.59 ± 0.12 ^a^	2625.08 ± 0.10 ^c^

C—carp roe salad without the addition of carrot powder, I1, and I2—carp roe salad with the addition of 6 and 12% (*w*/*w*) carrot powder. Mean values preceded by at least one common letter in the same row are not significantly different (*p* > 0.05).

**Table 3:** Phytochemical characteristics and antioxidant activity of carp roe salad samples with the addition of carrot powder.

Phytochemical Characterization	Samples of Carp Roe Salad		
	C	I1	I2
Total carotenoid content, mg/100 g DW	-	84.01 ± 3.39 ^b^	111.01 ± 1.68 ^a^
Total flavonoid content, mg CE/100 g DW	21.85 ± 3.06 ^c^	25.86 ± 3.74 ^b^	28.72 ± 2.70 ^a^
Total polyphenol content, mg GAE/100 g DW	66.18 ± 1.78 ^c^	68.86 ± 1.10 ^b^	88.91 ± 1.87 ^a^
Antioxidant activity, µM TE/g DW	528.16 ± 9.38 ^c^	550.66 ± 9.25 ^b^	588.32 ± 9.41 ^a^

C—carp roe salad without the addition of carrot powder, I1, and I2—carp roe salad with the addition of 6 and 12% (*w*/*w*) carrot powder. Mean values preceded by at least one common letter in the same row are not significantly different (*p* > 0.05).

**Table 4:** Colorimetric parameters of carp roe salad samples.

Samples of Carp Roe Salad	L*	a*	b*	Chroma	Hue Angle	ΔE
C	75.09 ± 0.02 ^a^	3.38 ± 0.07 ^c^	10.51 ± 0.03 ^c^	11.04 ± 0.06 ^c^	1.26 ± 0.022 ^b^	-
I1	66.78 ± 0.05 ^b^	3.96 ± 0.03 ^b^	15.57 ± 0.02 ^b^	16.07 ± 0.11 ^b^	1.32 ± 0.05 ^a^	9.75 ± 0.06 ^b^
I2	62.81 ± 0.01 ^c^	4.75 ± 0.02 ^a^	22.01 ± 0.01 ^a^	22.52 ± 0.24 ^a^	1.36 ± 0.05 ^a^	16.88 ± 0.13 ^a^

C—carp roe salad without the addition of carrot powder, I1, and I2—carp roe salad with the addition of 6 and 12% (*w*/*w*) carrot powder. Mean values preceded by at least one common letter in the same column are not significantly different (*p* > 0.05).

**Table 5:** Textural parameters of carp roe salad samples.

Textural Parameters	C	I1	I2
Firmness, N	0.21 ± 0.01 ^c^	0.38 ± 0.02 ^b^	0.52 ± 0.01 ^a^
Adhesion, mJ	2.48 ± 0.11 ^c^	3.99 ± 0.09 ^b^	4.25 ± 0.07 ^a^
Cohesiveness	0.11 ± 0.01 ^a^	0.09 ± 0.01 ^b^	0.07 ± 0.01 ^c^
Elasticity, mm	3.88 ± 0.12 ^a^	3.70 ± 0.11 ^b^	2.71 ± 0.08 ^c^

C—carp roe salad without carrot powder, I1 and I2—carp roe salad with 6 and 12% (*w/w*) carrot powder. Mean values preceded by at least one common letter in the same row are not significantly different (*p* > 0.05).

**Table 6:** Apparent viscosity of the carp roe salad samples measured at various shear rates over the stepped flow tests. C—carp roe salad without carrot powder, I1 and I2—carp roe salad with 6 and 12% (*w*/*w*) carrot powder.

	C	I1	I2
η (Pa·s) at 1 s^−1^	71.19 ± 2.49 ^c^	185.60 ± 6.50 ^b^	372.75 ± 17.46 ^a^
η (Pa·s) at 10 s^−1^	8.63 ± 0.17 ^c^	25.13 ± 0.52 ^b^	50.99 ± 2.44 ^a^
η (Pa·s) at 100 s^−1^	2.13 ± 0.09 ^b^	3.98 ± 0.02 ^a^	4.01 ± 0.03 ^a^

C—carp roe salad without carrot powder, I1 and I2—carp roe salad with 6 and 12% (*w*/*w*) carrot powder. Mean values on the same row accompanied by different superscript letters are significantly different at *p* < 0.05, based on the Tuckey method.

## Data Availability

The original contributions presented in the study are included in the article, further inquiries can be directed to the corresponding author.
